# Resourceful and economical designing of fermentation medium for lab and commercial strains of yeast from alternative feedstock: ‘transgenic oilcane’

**DOI:** 10.1186/s13068-025-02606-9

**Published:** 2025-01-31

**Authors:** Shraddha Maitra, Bruce Dien, Kristen Eilts, Nurzhan Kuanyshev, Yoel R. Cortes-Pena, Yong-Su Jin, Jeremy S. Guest, Vijay Singh

**Affiliations:** 1 Department of Agricultural and Biological Engineering, University of Illinois Urbana-Champaign, Urbana, IL 61801 USA; 2 United States Department of Agriculture (USDA), Bioenergy Research Unit, Agricultural Research Service (ARS), National Center for Agricultural Utilization Research (NCAUR), 1815 North University Street, Peoria, IL 61604 USA; 3 Carl R.Woese Institute for Genomic Biology, University of Illinois Urbana-Champaign, Urbana, IL 61801 USA; 4 Department of Civil and Environmental Engineering, University of Illinois Urbana-Champaign, Urbana, IL 61801 USA; 5 U.S. Department of Energy Center for Advanced Bioenergy and Bioproducts Innovation, University of Illinois Urbana-Champaign, Urbana, IL 61801 USA

**Keywords:** Fermentation media development, Fermentation, Drop-in-fuel, Lignocellulosic hydrolysate, Transgenic oilcane, C6/C5 metabolizing yeasts

## Abstract

**Background:**

Sugarcane plant engineered to accumulate lipids in its vegetative tissue is being developed as a new bioenergy crop. The new crop would be a source of juice, oil, and cellulosic sugars. However, limited tolerance of industrially recognized yeasts towards inhibitors generated during the processing of lignocellulosic biomass to produce fermentable sugars is a major challenge in developing scalable processes for second-generation drop-in fuel production. To this end, hydrolysates generated from engineered sugarcane—‘oilcane’ bagasse contain added phenolics and fatty acids that further restrict the growth of fermenting microorganisms and necessitate nutrient supplementation and/or detoxification of hydrolysate which makes the fermentation process expensive. Herein, we propose a resourceful and economical approach for growing lab and commercial strains of *S. cerevisiae* on unrefined cellulosic sugars aerobically and fermentatively.

**Results:**

An equal ratio of hydrolysate and juice was found optimum for growth and fermentation by lab and commercial strains of *Saccharomyces cerevisiae* engineered for xylose fermentation. The industrial strain grew and fermented efficiently under low aeration conditions having an ethanol titer, yield, specific and volumetric productivities of 46.96 ± 0.19 g/l, 0.51 ± 0.00 g/g, 0.27 ± 0.02 g/g.h and 1.95 ± 0.01 g/l.h, respectively, while the lab strain grew better under higher aeration conditions having the ethanol titer, yield, specific and volumetric productivities of 24.93 ± 0.09, 0.27 ± 0.00 g/g, 0.17 ± 0.00 g/g.h and 1.04 ± 0.00 g/l.h, respectively. Acclimation of cultures in a blended medium significantly improved the performance of the yeast strains.

**Conclusions:**

The addition of transgenic oilcane juice, which is inedible and rich in amino acids, to the hydrolysate averted the need for expensive nutrient supplementation and detoxification steps of hydrolysate. The approach provides an economical solution to reduce the cost of fermentation at an industrial scale for second-generation drop-in fuel production.

**Graphical Abstract:**

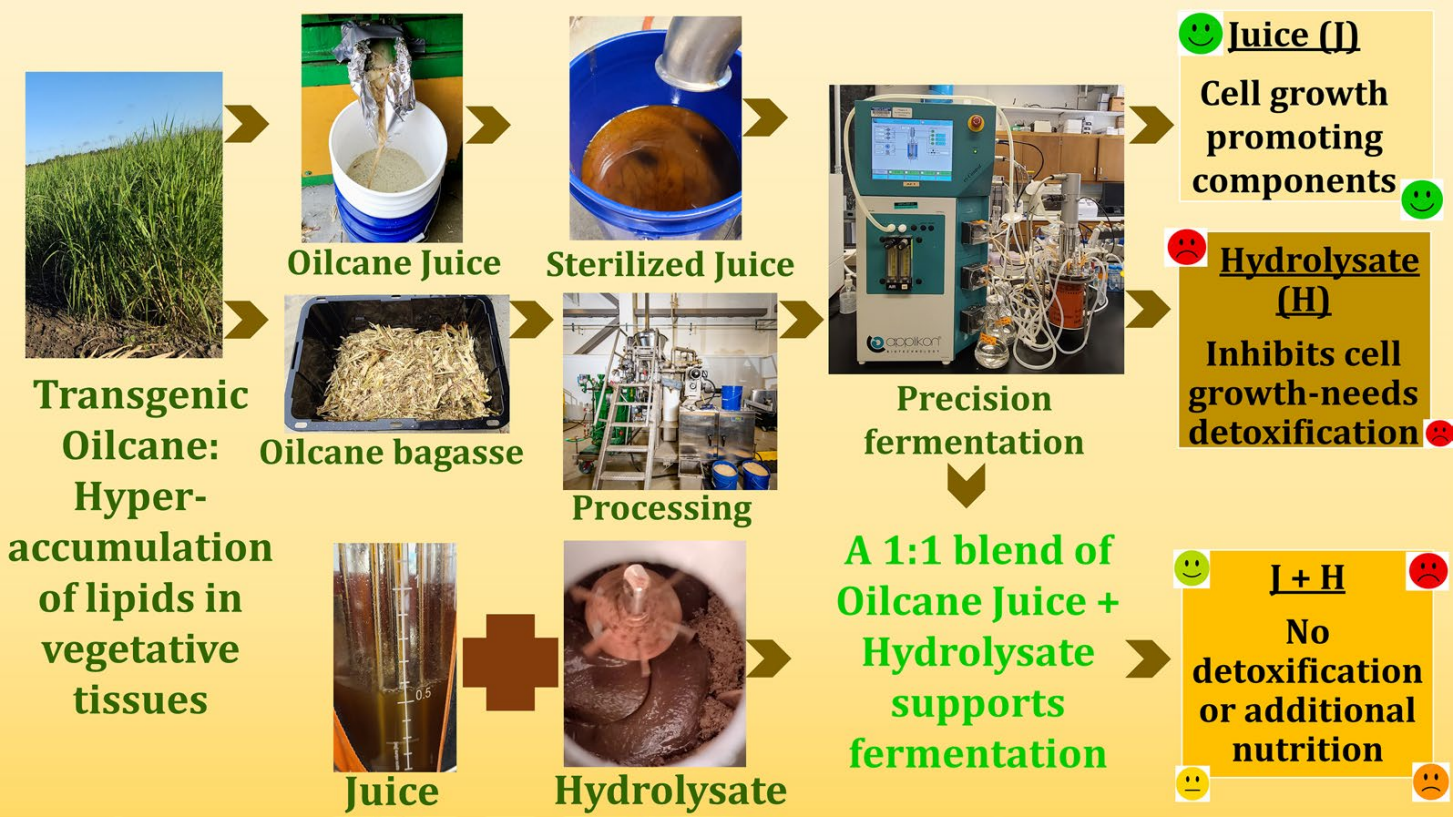

**Supplementary Information:**

The online version contains supplementary material available at 10.1186/s13068-025-02606-9.

## Introduction

Utilizing lignocellulosic biomass can reduce the use of fossil fuels and their corresponding environmental impacts. Currently, biofuels are produced globally using oilseeds, grains, and sugar crops. Their dual role as food or feed crops raises the concern that excess use of biofuels could inflate food costs. Past and continuing efforts to supplement these row crops with cellulosic sugars have been impeded by economics. To this end, a wide variety of new transgenic bioenergy crops with higher photosynthetic efficiencies and ability to grow in marginal lands are in development that also hyperaccumulate energy-dense molecules in the vegetative tissues, thereby, adding value and making them suitable as feedstocks for the production of drop-in-fuels and bio-based chemicals [1–5]. Typically, plants synthesize fatty acids and TAG molecules but do not hyperaccumulate in their vegetative tissues [6]. The warm-season bioenergy grasses such as sugarcane, sorghum, energycane, and miscan thus are being metabolically engineered to sequestrate carbon from juice to synthesize and hyperaccumulate triacylglyceride (TAG) molecules in their vegetative tissues [2, 4, 5]. The hyperaccumulation of vegetative lipids was achieved by coordinated upregulation and downregulation of multiple genes involved in TAG biosynthesis and catabolism, respectively [2]. In comparison to conventional bioenergy crops, these transgenic crops have the promise of producing both vegetative oil and cellulosic sugars that can be chemically or bio-converted to value-added bioproducts. Detailed techno-economic analyses by Huang et al. [7], Kumar et al. [8], and Yoel et al. [9, 10] reported that due to high biomass yield per unit are of cultivable land, the transgenic bioenergy crops are highly competitive and economical for environmentally sustainable production of biofuels and bio-based products [7–10]. The lipid hyperaccumulating transgenic line of sugarcane is referred to as ‘oilcane’ and has been used in the study for the development of a process/fermentation medium to produce biofuels and bioproducts.

The chemical-free processing of transgenic bioenergy crops has been successfully established at lab and a commercially relevant scale for the recovery of cellulosic sugars and vegetative lipids [11, 12]. Once the juice is extracted from oilcane stems, the residual lignocellulosic bagasse is pretreated and enzymatically hydrolyzed to recover fermentable sugars. However, GC/MS and LC/MS/MS analysis of untreated and pretreated transgenic oilcane bagasse showed that while chemical-free hydrothermal pretreatment preserves the lipids, it increases the amount of phenolic compounds significantly [13]. The lipids remain primarily with the residual biomass solids after pretreatment and enzymatic hydrolysis, where they can be conveniently extracted from residual solids. The process recovers ≥ 85% of cellulosic sugars and 55–88% of vegetative lipids [11, 12].

Although cellulosic sugars can be recovered from transgenic bioenergy crops with ≥ 85% efficiency, the hydrolysate produced after enzymatic saccharification of pretreated oilcane bagasse either completely inhibits microbial growth or impairs productivity and yield [13]. Inhibitory chemicals include the usual sugar degradation byproducts and phenolics [14–16] as well as lipids and increased levels of plant metabolites and phenolics [13]. Yeast strains are better able to cope with these inhibitors when fed a rich medium (e.g., vitamins, yeast extract, and peptone). However, these ingredients are often expensive and are not recommended for scale-up operations. Alternately, detoxification steps add to costs from sugar losses, slow down the process, and add considerable equipment and/or regent costs [17, 18].

Growth of yeast cultures on hydrolysate and juice sugars obtained from transgenic oilcane without nutrient supplementation has not been previously reported. The presence of lipids and modified plant physiology is expected to change the composition of the juice and hydrolysate, which is hypothesized to induce changes in yeast growth and metabolism. While *S. cerevisiae* is grown fermentatively for ethanol production, recombinant strains are also considered a chassis for the production of chemicals developed through synthetic biochemistry in aerobic cultures. The goal of this study is to establish a route to utilize juice and cellulosic sugars from transgenic bioenergy crops for aerobic and fermentative yeast cultures. A detailed compositional analysis of the juice and lignocellulosic hydrolysate was performed to quantify sugars, nutrients, and inhibitory chemicals. As will be described it was discovered that the juice contains a high level of nutrients. It was hypothesized that blending the juice and hydrolysate would lead to better fermentation results and that the juice would serve as a complete nutrient source for the yeast. Three *S. cerevisiae* strains, all engineered for xylose metabolism, were evaluated in aerobic and fermentative cultures for growth on hydrolysate, juice, and blends of the two. Ultimately, this study demonstrates the direct fermentation of oilcane hydrolysates without supplementation with nutrients (vitamins, amino acids, yeast extract or peptone), and/or detoxification for the first time after the development of transgenic bioenergy crops.

## Materials and methods

### Cellulosic feedstock

An alternative feedstock which is transgenic oilcane accession 1566 was developed as reported by Parajuli et al. [2]. Transgenic oilcane was genetically engineered to hyperaccumulate lipid molecules in vegetative tissues. It was field grown by the Plant Science Research and Education Unit (PSREU; Citra, FL, USA), leaves and stems separated at the harvesting site and transported to the Integrated Bioprocessing Research Laboratory (IBRL) located at the University of Illinois Urbana-Champaign, IL, USA and stored under refrigerated conditions at −20 ºC until further analysis.

### Juice and hydrolysate production

The transgenic oilcane stems were thawed before juice extraction. To maximize the extraction of juice, the transgenic oilcane stems were sequentially passed through a pilot-scale juice extractor and fiber press. The oilcane juice was stored at −20 ºC. Since, juice contained a complex mixture of fine biomass particles and waxes from oilcane stem and clogged filters used for sterilization, so, the juice was sterilized by autoclaving at 121 ºC for 15 min.

The resulting bagasse was washed to remove soluble sugars that would otherwise be quickly converted to furans during the pretreatment step. The washed bagasse was dried, hammer-milled, and adjusted for moisture content before pretreatment. Chemical-free hydrothermal pretreatment was performed at 50% w/w biomass solids loading at 190 ºC for 10 min in a pilot scale continuous hydrothermal pretreatment reactor followed by disc milling. Transgenic oilcane stems were processed and pretreated at the pilot scale facility as reported previously [11]. The pretreated bagasse was saccharified enzymatically at 25%w/w solids loading for 72 h at 50 ºC in an orbital shaker incubator agitated at 180 rpm at lab-scale according to the modified standard protocol NREL/TP-5100–63351 [19]. The reaction mixture contained 5 mM citrate buffer, and enzyme (16.9 mg protein/g dry pretreated biomass) (NS 22257, Novozymes North America, Inc., Franklinton, NC, USA). Residual biomass was separated from the hydrolysate by centrifugation (2739 g for 10 min). Hydrolysate was sterilized by filtering through a 0.2 µm membrane. The hydrolysate and juice were each analyzed for sugars using HPLC.

### Microorganism and cultivation

Two genetically engineered lab strains of *S. cerevisiae* developed at the University of Illinois Urbana-Champaign, IL, USA (CT2 Pro and NKSW7-1) [20, 21] and one genetically engineered commercial strain (kindly provided by DSM (Heerlen, The Netherlands)) were used to evaluate the newly developed process media. The strains were grown on YPD20 solid medium containing 1% (w/v) yeast extract, 2% (w/v) Peptone, and 20 g/L of glucose supplemented with 2% agar and in YPD20 medium for growing primary cultures.

### Kinetic studies of lab strains

Kinetic studies of the engineered lab yeast strains (CT2 Pro and NKSW7-1) were performed using YPD40 (40 g/l of glucose), YPX40 (40 g/l of xylose), and YPD30X10 (30 g/l of glucose and 10 g/l of xylose) in shake flasks incubated in an orbital shaker incubator operating at 30 ºC and agitated at 200 rpm. Cultures were inoculated to a cell OD_600_ of approximately 0.1. Samples were obtained at regular intervals of 3 h and analyzed for sugar consumption, ethanol production, and cell biomass. Kinetic experiments were performed in triplicates. The commercial strain was proprietary protected; therefore, kinetic studies were not performed. Kinetics on complex media was used as the control strain for growth and fermentation experiments on oilcane hydrolysate and juice blends.

### Propagation of strains in different compositions of hydrolysate and juice media

Preliminary screening of growth for the engineered strains on hydrolysate and juice was performed at a culture-to-flask volume of 0.40 using 500 mL shake flasks. The strains were inoculated at an initial cell OD_600_ of ~ 1.0 and incubated in an orbital shaker incubator at 30 °C and 200 rpm. The composition of juice, hydrolysate, and juice + hydrolysate blended media with or without added nutrients is listed in Table 2. YPD20 cultures were included as a control. The cells were incubated for 10 days to accommodate the probability of a long lag period. The experiments were performed in duplicates.

Those juice and hydrolysate media that afforded better yeast growth (Fig. 3) were selected and tested under controlled temperature (30 °C), pH (5), aeration (50%), and mixing (1000 rpm) using a Biolector XT microbioreactor system (Beckman Coulter, Life Sciences). Cultures were inoculated with an OD_600_ of 0.5 into the Biolector micro-culture plates, which were equipped with in situ optical pH and DO probes. The microbioreactor enables continuous measurement of biomass, pH, dissolved oxygen, and agitation. Cell growth was monitored for 10 days. Media samples from microtiter wells were filtered and analyzed for sugar consumption. Experiments were run in triplicate.

### Bioreactor setup

A 1-L non-jacketed Applikon reactor system (ezControl, Applikon Biotechnology, The Netherlands) with 300 ml working volume was used for growth kinetic studies on undiluted hydrolysate and an equal hydrolysate and juice blend in a batch culture. The temperature was maintained at 30ºC and the pH at 5.5 by the automatic addition of 10 N NaOH/10 N H_2_SO_4_. The culture was aerated at 1 vvm. Dissolved oxygen levels were maintained at 50% saturation using cascade control of agitation followed by aeration. Antifoam (0.1% silicone oil) was used to control foaming. Cultures were sampled thrice daily for 7 days and samples were analyzed for cell biomass, sugars, ethanol, and other fermentation byproducts.

### Acclimation studies

Engineered *S. cerevisiae* strains were precultured in the final media composition to adapt them as reported by Shin et al. (2024) with modification [22]. The strains inoculated from glycerol stock were first cultured in 15 ml of YPD and incubated for 48 h at 30 °C and 200 rpm in an orbital shaker incubator. After 48 h, the cultures were inoculated in the sustainable media (1:1 hydrolysate and juice) and incubated at 30 °C and 200 rpm in an orbital shaker incubator for 72 h. A 5% (v/v) precultured acclimated strains were inoculated in a 50 ml flask with a 25 ml working volume of sustainable media. The fermentation was carried out for 96 h and samples were taken at a regular interval of 24 h and analyzed for cell biomass, sugar consumption, ethanol, and other byproducts production. Flasks were shaken at 100 and 200 rpm to vary aeration. Experiments were performed in triplicate.

### Cell biomass determination

Both hydrolysate and juice had dark brown color and particulate matter making it difficult to accurately measure OD_600_. Therefore, the cell dry weight was determined by centrifuging the culture using 2 ml microcentrifuge tubes, washing the pellet twice with distilled water followed by drying at 80 ºC until mass remained constant.

### Sugars and inhibitors analysis

The hydrolysate and juice were analyzed for mono and oligosaccharides and sugar degradation products (HMF and furfurals), glycerol and acetic acid using an HPLC system (Waters alliance E2695 Separation module, Waters Corporation, MA, USA) equipped with an RID (refractive index) detector and Bio-Rad Aminex HPX-87H column (Bio-Rad, Hercules, CA, USA). The column was maintained at 50 ºC and a 0.05 M H_2_SO_4_ mobile phase was used at a constant flow rate of 0.6 ml/min. Bio-Rad Aminex PPX-87P column was used at 80 ºC and a constant flow rate of 0.6 ml/min with high purity 18 MΩ deionized water as mobile phase for improved resolution of sucrose.

### Lipid, metabolite, and vitamin analysis

The hydrolysate and juice fractions were analyzed for amino acids, lipids and metabolites at the Metabolomics Lab, Roy J. Carver Biotechnology Center, University of Illinois Urbana-Champaign, IL, United States. Untargeted lipidomics were performed using an UHPLC–Orbitrap–MS. The samples were run with both positive and negative ionization modes to capture a wider range of lipids. SPLASH^®^ LIPIDOMIX^®^ surrogate internal standards and multiple instrument internal standards were used as representatives of different lipid classes, including BioIVT plasma to assess intra- and inter-batch variability. Identifications of compounds were made using the LipidBlast spectral database in combination with the in-house database generated using chemical standards. Untargeted Metabolomics was performed using UHPLC–Orbitrap–MS. Each sample was injected twice. Two different columns (reverse-phase and hydrophilic interaction liquid chromatography (HILIC)), mobile phase compositions, and ionization modes (positive and negative) were used. Metabolite spectra and corresponding retention times were confirmed using chemical standards to generate an in-house spectral library, NIST 20, MoNA, MassBank EU, and GNPS spectral libraries for metabolite identification. Semi-quantitative data in the form of peak height, adjusted for internal standards and relative fractions were obtained. Amino acids were quantified with a targeted method using LC–MS/MS with multiple reaction monitoring (MRM). Quantification was performed using calibration curves for 9 labeled internal standards at 10 concentration levels along with Standard reference material (SRM) 1950, which contains certified and reference values.

Juice and hydrolysate were obtained from one transgenic variety which was processed in one big batch at pilot-scale. Hence, only one replicate of oilcane hydrolysate and juice was used for an untargeted complete metabolite analysis for lipids and phenolics using LC–MS to evaluate the relative fraction of lipids and phenolics in the samples. However, the analyses for exact amounts of amino acids were performed in triplicates.

### Equations used for calculating the kinetics of yeast

The following points were considered during the calculations:

1. Total sugars in the transgenic oilcane hydrolysate and juice contain glucose, xylose, fructose, sucrose, arabinose and cellobiose. Arabinose was not consumed by the yeast strains.
2. For ethanol yield estimation, the highest ethanol titer was considered, and corresponding sugar consumption.
3. For productivity estimation, the highest biomass and ethanol titer were considered with their substrate consumption and fermentation time.
  1 $$Biomass\, yield \left( \frac{g}{g} \right) = \frac{{Cell\, dry weight \left( {g/l} \right)}}{{Sugar\, consumed \left( {g/l} \right)}}$$
  2 $$Ethanol\, yield = \frac{{Ethanol\, titer \left( {g/l} \right)}}{{Sugar\, consumed \left( {g/l} \right)}}$$
  3 $$Specific\, ethanol\, productivity \left( {\frac{g}{g.h}} \right) = \frac{{Ethanol\, titer \left( {g/l} \right)}}{{Cell\, dry\, weight \left( \frac{g}{l} \right).Fermentation\, time \left( h \right)}}$$
  4 $$Volumetric\, ethanol\, productivity \left( {\frac{g}{l.h}} \right) = \frac{{Ethanol\, titer \left( {g/l} \right)}}{Fermentation\, time \left( h \right)}$$
  5 $$Specific\, substrate\, consumption \left( {\frac{g}{g.h}} \right) = \frac{{Sugar\, consumed \left( {g/l} \right)}}{{Cell\, dry\, weight \left( \frac{g}{l} \right).Fermentation\, time \left( h \right)}}$$

### Statistical analysis

Experiments were performed in triplicates. Three replicates of each sample were used for analysis of variance was performed using Minitab Statistical Software (Minitab Inc., USA) with a confidence interval of 95% (*p* < 0.05). Oilcane juice was obtained from pilot scale processing in one batch.

## Results

### Compositions of hydrolysate and juice derived from transgenic oilcane

**Fig. 1:**
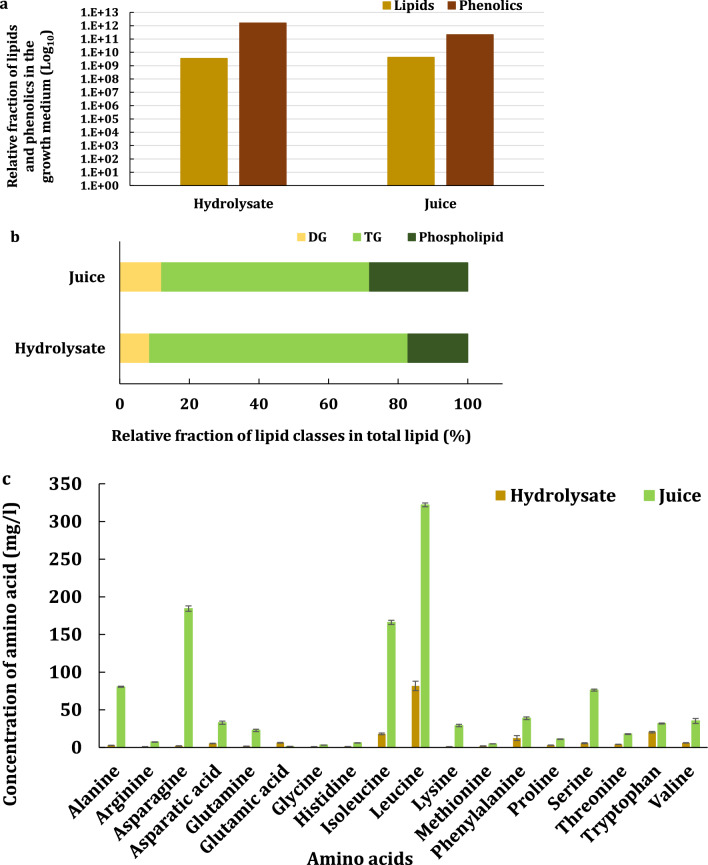
LC/MS analysis of (**a)** total lipids and metabolites, (**b)** various lipid classes [diglyceride (DG), triglyceride (TG) and phospholipids], and (**c)** LC–MS/MS analysis of 18 essential amino acids in juice and hydrolysate derived from transgenic oilcane

The hydrolysate obtained from pretreated oilcane bagasse contained monosaccharides (~ 128 g/l total sugars), lipids (TAGs, DAGs, and phospholipids), phenolics (metabolites and compounds released from lignin in response to pretreatment) and sugar degradation products, such as 5-hydroxymethyl furfurals (HMF) and furfurals (Fig. 1a, b, Tables 1 and S1). Trace amounts of amino acids were also observed in the hydrolysate (Fig. 1c) with a total concentration of 174 ± 14 mg/l (for the 18 essential amino acids). The concentrations of HMF, furfurals, and acetic acid generated during pretreatment were 0.19 ± 0.01 g/l, 0.15 ± 01, and 6.15 ± 0.02 g/l, respectively (Additional file Table S1). The oilcane juice contained 204 ± 6 g/l of soluble sugars including sucrose, fructose, and glucose (Table 1) along with lipids, phenolics, and amino acids (Fig. 1a–c). The concentrations of the 18 essential amino acids ranged from 5 to 322 mg/l.

**Table 1:** Concentrations of sugars in transgenic oilcane hydrolysate, juice and an equal blend of hydrolysate and juice used as media for ethanol fermentation

Growth media	Glucose (g/l)	Xylose (g/l)	Sucrose (g/l)	Fructose (g/l)	Arabinose (g/l)	Cellobiose (g/l)	Total fermentable sugar (g/l)
Hydrolysate	74.5 ± 2.12	42.57 ± 4.87	bdl^*^	3.72 ± 0.19	2.25 ± 0.21	4.96 ± 0.85	128.00 ± 11.96
Juice	17.20 ± 1.54	bdl^*^	174.56 ± 3.51	12.03 ± 1.14	bdl	bdl	203.79 ± 6.19
Hydrolysate + Juice (1:1)	52.10 ± 14.57	39.75 ± 10.92	34.64 ± 19.84	24.32 ± 4.03	bdl	3.32 ± 1.79	154.13 ± 51.15

^*^bdl—below detection limit

A qualitative LC/MS analysis showed that the relative fraction of phenolics in the hydrolysate was 10 times higher than that of the juice (Fig. 1a). However, the relative fractions of total lipids in hydrolysate and juice were similar. The relative percentage of phospholipids in juice was higher than in the hydrolysate (Fig. 1b). Phospholipids are considered growth promoting (Additional file Table 2a). Aminoacylated phospholipids have been reported to help bacteria adapt by altering the organization and function of the membrane–lipid domains [23, 24]. The lipid class analysis showed that both hydrolysate and juice contained mostly triglycerides followed by phospholipids and diglycerides. A major fraction of triglycerides had C16:0 and C18:0 side chains (Additional file Table 2b). A quantitative HPLC analysis of oilcane juice and hydrolysate showed that juice (1069.0 ± 23.9 mg/l of amino acid) had 6.15 times higher amino acids per unit volume than hydrolysate (173.9 ± 14.4 mg/l of amino acid). Leucine (322.0 ± 2.5 mg/l), asparagine (184.3 ± 3.6 mg/l), and isoleucine (166.1 ± 2.8 mg/l) were the four predominant amino acids in juice, while leucine (81.7 ± 6.2 mg/l) was main amino acid in hydrolysate followed by tryptophan (20.2 ± 1.0 mg/l) and isoleucine (18.0 ± 1.0 mg/l) (Fig. 1c). The juice contained significant amounts of amino nitrogen, which could recommend it as a complex nutrient source for fermentation.

### Growth and fermentation kinetics of engineered *S. cerevisiae* on complex media

**Fig. 2:**
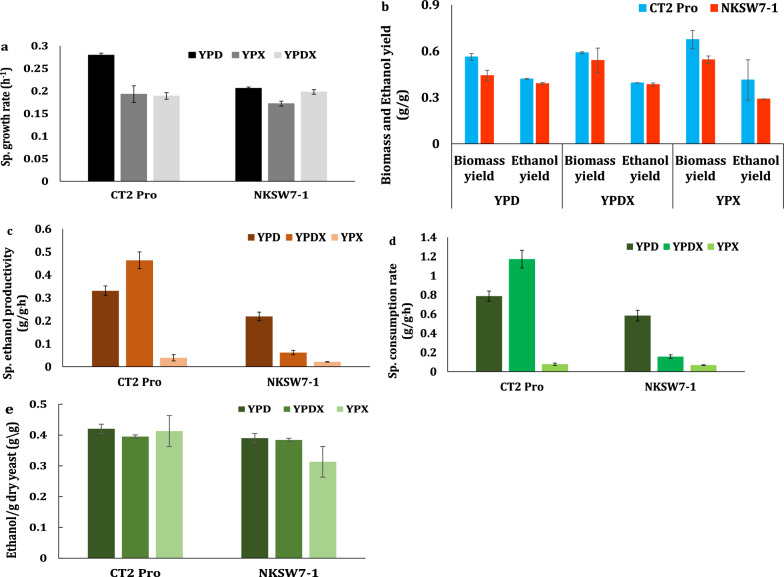
Kinetics of metabolically engineered *S. cerevisiae* strains CT2-Pro and NKSW7-1 (**a)** specific growth rates, (**b)** biomass and ethanol yields, (**c)** specific ethanol productivity, (**d)** specific glucose consumption rate and** (e) **ethanol yield per gram of dry cell biomass on complex medium (YP) containing glucose (YPD), xylose (YPX), and glucose + xylose (YPDX) as carbon source

Two laboratory strains (CT2 Pro and NKSW7-1) engineered to ferment C6/C5 sugars were tested for their growth and fermentation kinetics on a complex medium containing glucose, xylose, and a glucose + xylose mixture (3:1 as expected in hydrolysate) before analyzing their efficiencies in the new process/fermentation medium. CT2 Pro is a xylose-utilizing *S. cerevisiae* strain constructed from strain SR8 using Cas9, guide RNAs, and expression cassettes containing xylose metabolizing genes (*XYL1*, *XYL2*, and *XYL3*). Strain CT2 Pro does not contain any markers of bacterial origin making it an excellent candidate for commercial use [20]. On the other hand, NKSW7-1 was developed by deleting the major hexose transporters of *S. cerevisiae* and expressing AtSWEET7, a fungal non-specialized monosaccharide transporter. This enables NKSW7-1 to simultaneously ferment glucose, xylose, fructose, and mannose whether present in synthetic medium, hydrolysate, and sugarcane juice [21, 25]. The growth kinetics of the two laboratory strains were studied to learn their maximum yields, rates, and productivities on a complex nutrient medium supplemented with refined sugars (Fig. 2).

The maximum specific growth rates of CT2 Pro on glucose, xylose, and mixed sugar (glucose + xylose) were 0.28 ± 0.004 h^−1^, 0.19 ± 0.02 h^−1^, and 0.18 ± 0.01 h^−1^, respectively. Strain NKSW7-1 grew 1.4 and 1.1 times slower on glucose (0.20 ± 0.002 h^−1^) and xylose (0.17 ± 0.01 h^−1^) than CT2 Pro at a similar rate on mixed sugars (0.19 ± 0.01 h^−1^; Fig. 2a). In aerobic cultures, both CT2 Pro and NKSW7-1 showed theoretical maximum biomass yields on glucose, xylose, and mixed sugar media (Fig. 2b). Complete sugar consumption was observed within 48 h for CT2 Pro cultures and 72–96 h for the NKSW7-1 cultures. Ethanol yields of CT2 Pro on glucose, xylose, and mixed sugars were 0.42 ± 0.003 g/g, 0.41 ± 0.13 g/g, and 0.40 ± 0.001 g/g, respectively. NKSW7-1 had 1.07 times and 1.4 times lower ethanol yields on glucose and xylose and a similar yield on mixed sugar compared to CT2 Pro (Fig. 2b). CT2 Pro showed the maximum specific ethanol productivity on mixed sugars (0.46 ± 0.03 g/g.h) followed by glucose and xylose as the sole carbon source, while NKSW showed the highest specific ethanol productivity on glucose (0.21 ± 0.01 g/g.h) followed by xylose and mixed sugar media (Fig. 2d). As expected, specific sugar consumption rates mirrored specific ethanol productivities. CT2 Pro had 1.3 times, 1.1 times, and 7.8 times higher specific sugar consumption rates on glucose, xylose, and mixed sugar media as compared to NKSW7-1, respectively (Fig. 2d). Notably, both strains had similar ethanol yield per gram dry cell biomass when fermenting glucose, xylose, or mixed sugars (Fig. 2e).

### Fermentations of juice and hydrolysate mixtures with and without supplemental nutrients

Yeast strains CT2 Pro and NKSW7-1 were grown on various ratios of hydrolysate and juice and supplemental nutrients to determine their preferred medium (Table 2). Growth was measured after 120 h by measuring optical density. None of the cultures showed strong growth on hydrolysate that was undiluted or diluted with water/juice. However, when the pH was adjusted up to 5, the yeast strains grew well in hydrolysate diluted with either water or juice. In contrast, the yeast grew well on juice without pH adjustment.

**Table 2:** Growth of xylose-fermenting *S. cerevisiae* CT2 Pro and NKSW strains on various carbon sources with/without nutrient supplement

Media	Liquid ratios	CT2 pro growth	NKSW growth
Hydrolysate	100	−	−
Hydrolysate + Water	50:50	+	−
Hydrolysate + Water	20:80	+	+
Hydrolysate + Juice	50:50	+ +	+ +
Hydrolysate + Water + Juice	25:25:50	+ +	+ +
Juice	100	+ + + +	+ + + +
Hydrolysate + Water + urea^a^	50:50	−	−
Hydrolysate + Water + CSM^b^	50:50	−	−
Hydrolysate + Water + CSM^b^ + sodium sulfite^c^	50:50	−	−
Hydrolysate (pH 5)	100	+ +	−
Hydrolysate + Water (pH 5)	50:50	+ + + +	+ + + +
Hydrolysate + Juice (pH 5)	50:50	+ + + +	+ +

Cells were rated for growth: strong growth (+ + + +), medium growth (+ +), slight growth ( +) and no growth (−)

^a^Urea (5 g/l)

^b^Complete amino acids mixture (0.67 g/l)

^c^Sodium sulfite (12.5 mM/1.57 g/l)

**Fig. 3:**
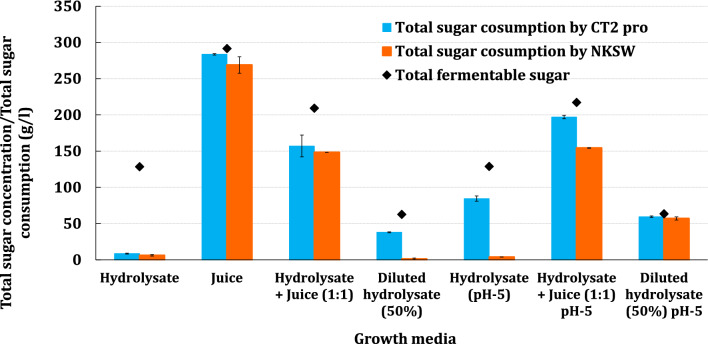
Aerobic growth of engineered xylose fermenting *S. cerevisiae* strains CT2-Pro and NKSW7-1 for 7 days in hydrolysate (100%), juice (100%), hydrolysate: juice (50:50%) and 50% dilute hydrolysate with/without pH adjustment. Diamonds and bars represent total sugar concentrations in the medium and total sugar consumption by yeast strains, respectively

Select conditions were repeated using the BioLector XT Microbioreactor, which is a microplate-based bioreactor system equipped with dissolved oxygen (DO) control and pH monitoring. Cultures were grown with high aeration (> 50% statured dissolved oxygen) in media that had either been raised to pH 5.0 or kept acidic. The carbon sources were undiluted hydrolysate or juice and 50% diluted hydrolysate, and an equal mixture of juice and hydrolysate (Fig. 3). CT2 Pro grew and consumed > 80% of total sugars in all the cultures except 100% hydrolysate without pH adjustment. CT2 Pro consumed all the sugars when cultured on oilcane juice and pH-adjusted hydrolysate diluted 50% with either juice or water. NKSW7-1 consumed all the sugars when growing on juice and pH-adjusted 50% diluted hydrolysate with water. NKSW7-1 did not grow on undiluted hydrolysate regardless of pH. NKSW7-1 grew on hydrolysate diluted 50% with either water or juice and consumed > 70% of the total sugars.

In summary, pH adjustment to 5 and dilution of hydrolysate either with water or juice are necessary for strong yeast growth. The results for the equally blended juice and hydrolysate are particularly notable, because the juice is rich in nutrients that support better fermentation efficiency, and avoids the cost of adding expensive growth nutrients.

### Growth of yeast strains on 100% hydrolysate and a 50:50 hydrolysate and juice blend

**Fig. 4:**
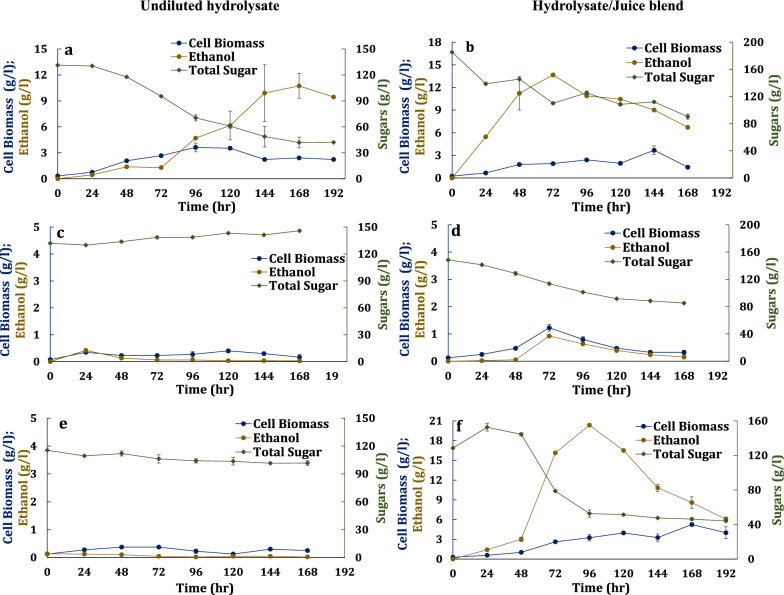
Growth and fermentation profile of metabolically engineered *S. cerevisiae* strains CT2-Pro (**a**, **b**), NKSW7-1 (**c**, **d**) and commercial yeast (**e**, **f**) on undiluted hydrolysate (**a**, **c**, **e**) and 50:50 Hydrolysate: Juice (**b**, **d**, **f**) derived from transgenic oilcane, respectively

The three yeast strains (CT2 Pro, NKSW7-1, and the commercial strain) were grown using a computer-controlled bioreactor maintained at ~ 50% DO levels to fully characterize aerobic growth. The yeast strains were grown on undiluted hydrolysate and hydrolysate blended 1:1 with juice, all adjusted to pH 5 (Table 3 and Fig. 4). Only CT2 Pro grew on undiluted hydrolysate. No growth was observed for NKSW7-1and the commercial yeast strain on undiluted hydrolysate. Though CT2 Pro grew on the undiluted hydrolysate, it had difficulty as evidenced by the 48 h lag time. It consumed approximately 67% of the provided sugars (88.54 ± 6.50 g/l). In the end, it did begin to consume xylose (Figure S1). As expected, ethanol yields were low and probably associated with the Crabtree effect (Fig. 4 and Additional file Figure S1).

**Table 3:** Kinetics of engineered *S. cerevisiae* strains CT2-Pro, NKSW7-1 and commercial strain on the transgenic oilcane hydrolysate and an equal blend of hydrolysate and juice used as media without detoxification or nutrient supplementation

*S. cerevisiae* strains	Carbon Source^a^	Total sugar (g/l)	Sugar consumed^b^ (g/l)	Ethanol titer (g/l)	Biomass yield (g/g)	Ethanol yield (g/g)	SEP^c^ (g/g^.^h)	VEP^d^ (g/l^.^h)	SSC Rate^e^ (g/g^.^h)
CT2-Pro	Hydrolysate	131.29 ± 0.01	88.54 ± 6.50	10.73 ± 1.42	0.027 ± 0.002	0.121 ± 0.007	0.026 ± 0.004	0.06 ± 0.008	0.22 ± 0.01
	50:50 H:J	185.89 ± 0.38	95.11 ± 3.32	13.66 ± 0.13	0.025 ± 0.001	0.181 ± 0.003	0.100 ± 0.009	0.19 ± 0.001	0.55 ± 0.04
NKSW7-1	Hydrolysate	132.02 ± 0.48	0	0	ND^f^	ND	ND	ND	ND
	50:50 H:J	148.63 ± 0.08	63.35 ± 0.55	0.92 ± 0.01	0.035 ± 0.003	0.026 ± 0.001	0.011 ± 0.001	0.01 ± 0.001	0.31 ± 0.03
Commercial strain	Hydrolysate	115.84 ± 0.26	0	0	ND	ND	ND	ND	ND
	50:50 H:J	152.67 ± 4.41	82.24 ± 0.32	20.33 ± 0.18	0.044 ± 0.009	0.273 ± 0.013	0.067 ± 0.011	0.21 ± 0.001	0.24 ± 0.05

^a^Carbon source consisted of either undiluted hydrolysate (H) or an equal blend of hydrolysate and juice (H:J)

^b^Total sugar consumption includes consumption of only fermentable sugars (arabinose is not included) till 168 h

^c^Specific ethanol productivity

^d^Volumetric ethanol productivity

^e^Specific sugar consumption rate

^f^Not determined—no growth was observed for NKSW7-1 and commercial yeast strain in hydrolysate cultures

For the equally blended hydrolysate and juice medium cultures, CT2 Pro, NKSW7-1 and the commercial strain consumed 48%, 57%, and 46% of the sugars within 168 h. No net cell growth was detected for NKSW7-1, and therefore, the sugar consumed was directed towards cell maintenance. The biomass yield of lab strain CT2 Pro was 1.76 times lower than the commercial strain. Both the lab strains and commercial strains utilized glucose and xylose sequentially; however, co-consumption of glucose and xylose was observed for NKSW7-1 (Additional file Figure S1). NKSW7-1 also produced 0.31 ± 0.01 g/l of xylitol, which is an intermediate metabolite of xylose consumption (Additional file Table S1). In addition, acetic acid was one of the inhibitory compounds present in the hydrolysate and juice medium which could have a deleterious effect on the cellular metabolism. It was noted that acetic acid concentrations in the fermentation medium decreased at the end of alcoholic fermentation for the lab yeast strains indicating uptake of acetate as a carbon source by the engineered lab yeast strains (Additional file Table S1). This is not unexpected for *S. cerevisiae* cells to uptake the extracellular acetic acid at a pH around the pKa of acetic acid (4.76). At pKa below 4.76, acetic acid remains as CH_3_COOH, in undissociated form, and diffuses passively across the cell membrane. On the other hand, the intracellular pH remains well above the pKa of acetic acid, so, the anion accumulates in the cell causing increased oxidative stress [26].

In summary, the equal blend of juice and hydrolysate medium supported the growth of both lab and commercial yeast strains. The next step was process optimization to improve the fermentation efficiencies of the yeast strains.

### Acclimating yeast improved fermentation efficiencies

In the previous experiment, none of the yeast growing on the blended sugar mixture exhausted the sugars by 8 days (Fig. 4). This is likely caused by osmatic stress from the high sugar concentration and inhibitors (e.g., phenolics and lipids). Osmotic stress was lowered by diluting the blended sugars ~ 120 and ~ 75 g/l sugar. Inhibitor stress was reduced by growing the seed cultures on hydrolysate and juice medium to acclimate the cells. Due to the limited availability of the transgenic oilcane hydrolysate and juice, these experiments were performed at smaller volumes in shake flask instead of the bioreactor.

**Fig. 5:**
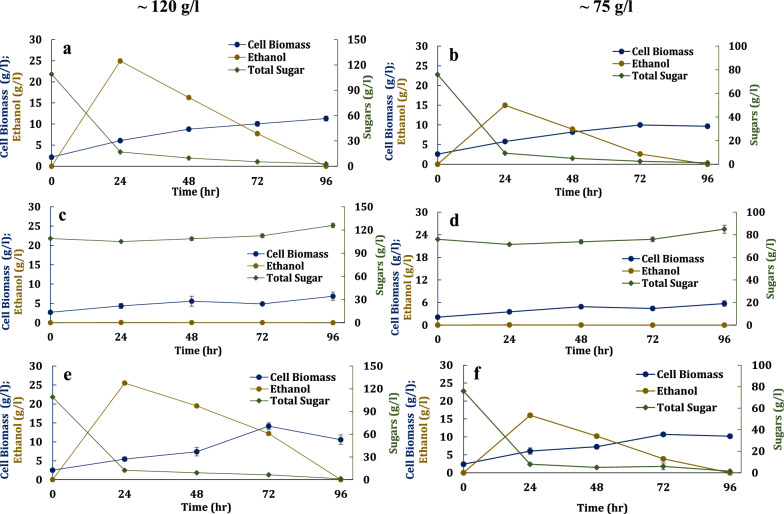
Growth and fermentation profile of metabolically engineered *S. cerevisiae* strains CT2-Pro (**a**, **b**), NKSW7-1 (**c, d**) and commercial yeast (**e**, **f**). Equal blended hydrolysate and juice media were diluted to achieve 120 g/l and 75 g/l of total sugars. Seed cultures were grown on blended hydrolysate and juice media for acclimatization

**Fig. 6:**
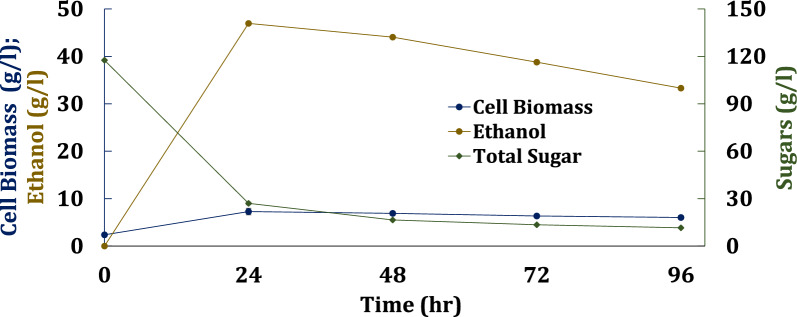
Fermentations of an equal blend of transgenic oilcane hydrolysate and juice using xylose-fermenting commercial *S. cerevisiae* strain. Cells were acclimated by growing the seed on the blended sugars

Both CT2-Pro and the commercial yeast strains that were acclimated grew on the blended sugars at both concentrations in aerobic cultures (e.g., incubator shaker set at 200 rpm) (Figs. 5 and 6, Additional file Figure S2). In contrast to the prior experiment, both yeast strains exhausted the sugars within 96 h. The acclimated cultures grew and fermented more efficiently in the hydrolysate and juice media than the unacclimated yeast cultures (Table 4, Additional file Table S3, Figure S3). For instance, the unacclimated commercial yeast cultures consumed approximately 10% of the total sugar and had a 48 and 72-h lag time when grown on the ~ 120 and ~ 75 g/l sugar blend, respectively (Additional file Figure S3e, f). However, neither the acclimated nor unacclimated NKSW7-1 cultures grew (Fig. 5c, d, Additional file Figure S3c, d). The biomass yields for CT2-Pro and the commercial yeast strain were 56–87 mg/g sugar and were higher for the ~ 75 g/l blended sugars, perhaps because the medium with lower sugar concentration had better exchange of gas (aeration) (Table 4). However, the specific sugar consumption rate was higher for ~ 120 g/l which were 0.63 gg^−1^ h^−1^ and 0.75 gg^−1^ h^−1^ for CT2 Pro and commercial strain, respectively. This is because, at higher sugar concentrations, the culture conditions were oxygen-limited and the excess sugar was channelized towards ethanol fermentation rather than respiratory growth [27]. For the acclimated cultures, most of the sugars were consumed within 24 h (Additional file Figure S.2).

**Table 4:** Kinetics of acclimated cultures of engineered *S. cerevisiae* strains CT2-Pro, NKSW7-1 and commercial strain on an equal blend of transgenic oilcane hydrolysate and juice used as media without detoxification or nutrient supplementation

*S. cerevisiae* strains	Total Sugar concentration (g/l)	Aeration condition^a^	Ethanol titer (g/l)	Biomass yield (g/g)	Ethanol yield (g/g)	SEP^b^ (g/g^.^h)	VEP^c^ (g/l^.^h)	SSC^d^ rate (g/g^.^h)
CT2-Pro	109.02 ± 0.14	Aerated	24.93 ± 0.09	0.065 ± 0.001	0.27 ± 0.002	0.17 ± 0.001	1.04 ± 0.004	0.63 ± 0.007
	75.98 ± 0.025	Aerated	14.96 ± 0.06	0.087 ± 0.001	0.22 ± 0.002	0.11 ± 0.001	0.62 ± 0.002	0.48 ± 0.004
	117.53 ± 0.23	Fermentative	0	ND^e^	ND	ND	ND	ND
NKSW7-1	109.02 ± 0.14	Aerated	0	ND	ND	ND	ND	ND
	75.98 ± 0.025	Aerated	0	ND	ND	ND	ND	ND
	117.53 ± 0.23	Fermentative	0	ND	ND	ND	ND	ND
Commercial strain	109.02 ± 0.14	Aerated	25.51 ± 0.11	0.056 ± 0.005	0.26 ± 0.001	0.19 ± 0.017	1.06 ± 0.004	0.75 ± 0.06
	75.98 ± 0.025	Aerated	16.01 ± 0.14	0.089 ± 0.013	0.24 ± 0.002	0.11 ± 0.018	0.66 ± 0.005	0.48 ± 0.07
	117.53 ± 0.23	Fermentative	46.96 ± 0.19	0.080 ± 0.006	0.51 ± 0.001	0.27 ± 0.02	1.95 ± 0.014	0.52 ± 0.04

^a^Aerated and fermentative conditions refer to shaking at 200 rpm and 100 rpm, respectively

^b^Specific ethanol productivity

^c^Volumetric ethanol productivity

^d^Specific sugar consumption

^e^Not determined, because no growth was observed for these cultures

Finally the yeast strains were grown fermentatively on the ~ 120 g/l blended sugar at lower aeration conditions by operating the incubator shaker at 100 rpm to observe any improvement in the fermentation kinetics (Fig. 6, Table 4). As before, the seed cultures were acclimated by growing them on a blend of juice and hydrolysate. In this case, neither of the laboratory strain grew. The observation suggests the engineered lab yeast are less robust to inhibitor than the commercial strain under fermentative conditions. The commercial strain grew and used almost all of the sugars within the first 24 h. The maximum ethanol yield of 0.51 ± 0.001 g/g was achieved within 24 h with a maximum ethanol titer of 47 g/l on mixed sugars.

## Discussion

### Transgenic oilcane can be used for dedicated and sustainable supply of cellulosic sugars for biofuels and biobased products

Presently, food crops such as corn, sugarcane, and beetroot are the major contributors to the production of bio-based chemicals. Transgenic oilcane and energycane are being developed as new bioenergy crops that combine the advantages of oil, sugar, and fiber crops. These are determined to be a suitable crop for producing second-generation ethanol and renewable/bio-diesel. Transgenic crops are meant to grow on marginal land which could potentially allow a dedicated supply of oil and fermentable sugars to meet the increasing demand for biobased chemicals without competing for food or agricultural land [28]. Realizing these advantages will require developing novel processing technology. Recently, transgenic bioenergy crops, i.e., oilcane and transgenic energycane have been successfully processed at lab and industrially relevant scales to recover vegetative lipids and cellulosic sugars that can directly be bioconverted into a wide variety of bioproducts [11, 12]. However, the hydrolysates obtained from these transgenic crops post-saccharification contain significantly higher levels of phenolics, lipids, and metabolites as compared to non-transgenic bioenergy crops, i.e., sugarcane and sorghum, which restricts the growth and lowers the yields of fermentation microorganisms [29]. The study contributes one step forward towards the commercialization of alternative lignocellulosic biomass by demonstrating the development of an economical process/fermentation media without expensive nutrient supplementation or detoxification steps and its efficient fermentation by engineered lab and commercial strains of yeast.

Sugarcane juice is rich in nutrients and contains approximately 13–15% sucrose, 10–21% non-reducing sugar, 0.3–3% reducing sugars, 0.5–1% organic compounds, 0.5–1% nitrogenous compounds, 10–15% fibers, and 0.2–0.6% inorganic minerals [30]. Since oilcane is a genetically engineered variety of sugarcane, the oilcane juice is expected to have a similar nutrient profile. Juice from transgenic oilcane contained all the necessary amino acids needed for yeast growth (Fig. 1c). Unlike sugarcane juice, oilcane juice has not been approved (yet) for a food or feed market, which favors its utilization as a nutrient supplement for fermentation. It was found to be ideal for blending with cellulosic sugars, because it eliminated the need for supplemental nutrients and provided a source of sugars while diluting out the inhibitors. The blending of oilcane juice and bagasse hydrolysates aided in optimizing the development of an economic and sustainable fermentation medium. All the blends of hydrolysate to juice worked except for ratios < 1:1. A 1:1 ratio of undetoxified hydrolysate and juice (with no added nutrients) was found to be a complete growth medium for both the lab and commercial yeast strains. A minimum of equal blend of hydrolysate and juice media reported in this study averts the need to add yeast extract and peptone to initiate the growth of culture as often reported by fermentation studies using lignocellulosic hydrolysates. Thereby, allowing the yeast to grow on the blended sugars without requiring an inhibitor removal step. It is envisioned that these advances will favor the industrial use of oilcane for the production of biofuels and bio-based chemicals.

### Improving growth and fermentation efficiency of fermenting microbes on lignocellulosic hydrolysate

One of the major challenges in the utilization of lignocellulosic biomass is the process economics at the industrially relevant scale. The technoeconomic and life cycle assessments reported the competitiveness of transgenic crops for commercial economics and environmental sustainability for the production of biobased chemicals [7–9, 31]. However, the process simulations assume 80–90% of theoretical ethanol yield from fermentation on unrefined hydrolysate without nutrient supplementation, which is challenging to achieve experimentally. Hydrolysate produced from lignocellulosic biomass post chemical or hydrothermal pretreatment contains sugar degradation products such as HMF and furfurals which are inhibitory to the fermenting microbes [32, 33]. Detoxification of hydrolysates using filtration systems, resins, solvent extraction, or activated charcoal in addition to nutrient supplementation for fermenting microorganisms increases the production cost [32]. In addition to pretreatment inhibitors, the transgenic bioenergy crops contain enhanced levels of phenolics and lipids (present study and [13]). Authors’ previous study showed that untreated oilcane bagasse has significantly lower amounts of phenolics as compared to pretreated biomass [13] but still, the levels of phenolics in untreated transgenic crops are 10–100 times higher than non-transgenic crops, such as miscanthus and bioenergy sorghum [29]. Removal of phenolics needs additional processing of the hydrolysate which adds to the process cost. Note that a detailed techno-economic feasibility study by Cortés-Peña et al. [9] demonstrated higher economic sustainability and less market-driven sensitivity for co-fermentation of oilcane juice and hydrolysate due to improved ethanol and biodiesel production as compared to conventional feedstock and fermenting juice and hydrolysate separately [9]. The simulated sustainable co-fermentation process was experimentally proved in the study which was to develop a fermentation medium using oilcane hydrolysate and juice for a wide variety of microorganisms without adding to the cost of the process.

Herein the medium was evaluated using three engineered strains of *S. cerevisiae,* i.e., two lab strains (CT2 Pro and NKSW7-1) and one commercial strain. The CT2 Pro strain was constructed by applying the Cas9/CRISPR technique to improve xylose utilizing strain SR8 by inserting the *S. stipitis* XYL123 cassette. CT2 Pro strain is marker-free, enabling it to be used for industrial bioethanol production [20]. On the other hand, NKSW7-1 was constructed by replacing major hexose sugar transporters in engineered *S. cerevisiae* with non-selective fungal SWEET7 transporters [21, 25]. The C5/C6 fermenting commercial strain was kindly provided by DSM (Heerlen, The Netherlands). Each strain had definite characteristics and requirements. The fermentation of lab and commercial yeast strains grew in the optimized medium. CT2 Pro and commercial strains grew in both aerobic and fermentative cultures, while NKSW7-1 only grew in aerobic culture. The yeast strains (two lab strains and one obtained from bioethanol industry) were chosen purposefully to analyze the applicability of the fermentation medium on a broad scale. The NKSWS-7 lab strain did not perform well. However, the CT2 Pro lab strain and the commercial strain (from industry) were competitive and showed ethanol yields of 0.27 g/g and 0.51 g/g, respectively, in media containing high sugar concentrations (100–120 g/l) after adaptation. Further research is warranted to investigate if the blending of oilcane juice and cellulosic hydrolysate will support the growth of other yeast species or possibly bacteria for the production of other chemicals.

Acclimating the seed culture on dilute hydrolysate is recommended to achieve optimal ethanol yield and productivity. Fermenting microorganisms, specifically *S. cerevisiae* respond to their changing environment, such as high sugar, acetic acid, and ethanol concentrations by adjusting their cellular metabolism [23, 34–36]. In response to the environmental osmotic stress, yeasts express various stress-responsive transcriptional factors and genes to modulate their membrane fluidity [35]. Another way of achieving high ethanol yield and productivity in mixed sugar media is using a microbial consortium. Microbial consortia enable the modulation of the conversion rates for each sugar by tuning and optimizing the time of addition of each strain to control the overall process dynamics and minimize the burden of the fermenting microorganisms [22]. Engineered yeasts and microbial consortia capable of fermenting mixed sugars have been successfully developed [22, 37].

## Conclusion

The development of transgenic oilcane is potentially a large step forward for bio-based products, because it has higher oil productivity than commercial oil seed crops and higher carbon sequestration per unit of land. In addition to oil, the plant produces juice and cellulosic sugars. The study successfully demonstrates that the addition of nutrient-rich juice improves the growth and fermentation efficiencies of both laboratory and commercial strains of *S. cerevisiae* on hydrolysate, and hence acts as a microbiological medium supplement. The proposed approach avoids the requirement of additional nutrients or detoxification of the cellulosic hydrolysate.

## Supplementary Information

Supplementary material 1: Table 1: Concentrations of minor chemicals present in growth media constructed using transgenic oilcane hydrolysate and juice blends prior to and post-fermentation. Table 2: a) Phospholipids in oilcane hydrolysate and juice obtained using untargeted LC–MS metabolite analysis. The results are reported as signal's peak area counts normalized to the internal standard's peak area count and/or sample volume/sample weight. b) LC/MS analysis of lipid classes, lipid ions and ion formulae of ten major lipids in hydrolysates and juice derived from transgenic oilcane. Table 3: Growth kinetics of xylose metabolizing *S. cerevisiae* strains in aerobic cultures without acclimated strains. The medium was an equal blend of hydrolysate and juice. Fig. S1: Sugar consumption profile of metabolically engineered *S. cerevisiae* strains CT2-Pro, NKSW7-1and commercial yeaston 100% hydrolysateand 50:50 Hydrolysate: Juicederived from transgenic oilcane, Fig. S2: Sugar consumption profile of metabolically engineered *S. cerevisiae* strains CT2-Pro, NKSW7-1and commercial yeast. The strains were acclimated on 50:50 Hydrolysate: Juice media prior to fermentation. Hydrolysate: Juice 50:50 media was diluted to get a total sugar concentration of ~ 120 g/land ~ 75 g/l. Fig. S 3: Growth and fermentation profile of metabolically engineered *S. cerevisiae* strains CT2-Pro, NKSW7-1and commercial yeast. The strains were not acclimated prior to fermentation. Hydrolysate: Juice 50:50 media was diluted to get a total sugar concentration of ~ 120 g/land ~ 75 g/l.

## Data Availability

No data sets were generated or analysed during the current study.
