# Supplementary material for: Resourceful and economical designing of fermentation medium for lab and commercial strains of yeast from alternative feedstock: ‘transgenic oilcane’
Source: Biotechnol Biofuels Bioprod. 2025 Jan 31;18:14. doi: 10.1186/s13068-025-02606-9 (PMC11786580; doi:10.1186/s13068-025-02606-9)
Supplement: Supplementary file 1 — Supplementary material 1: Table 1: Concentrations of minor chemicals present in growth media constructed using transgenic oilcane hydrolysate and juice blends prior to and post-fermentation. Table 2: a) Phospholipids in oilcane hydrolysate and juice obtained using untargeted LC–MS metabolite analysis. The results are reported as signal's peak area counts normalized to the internal standard's peak area count and/or sample volume/sample weight. b) LC/MS analysis of lipid classes, lipid ions and ion formulae of ten major lipids in hydrolysates and juice derived from transgenic oilcane. Table 3: Growth kinetics of xylose metabolizing S. cerevisiae strains in aerobic cultures without acclimated strains. The medium was an equal blend of hydrolysate and juice. Fig. S1: Sugar consumption profile of metabolically engineered S. cerevisiae strains CT2-Pro, NKSW7-1and commercial yeaston 100% hydrolysateand 50:50 Hydrolysate: Juicederived from transgenic oilcane, Fig. S2: Sugar consumption profile of metabolically engineered S. cerevisiae strains CT2-Pro, NKSW7-1and commercial yeast. The strains were acclimated on 50:50 Hydrolysate: Juice media prior to fermentation. Hydrolysate: Juice 50:50 media was diluted to get a total sugar concentration of ~ 120 g/land ~ 75 g/l. Fig. S 3: Growth and fermentation profile of metabolically engineered S. cerevisiae strains CT2-Pro, NKSW7-1and commercial yeast. The strains were not acclimated prior to fermentation. Hydrolysate: Juice 50:50 media was diluted to get a total sugar concentration of ~ 120 g/land ~ 75 g/l. [file 13068_2025_2606_MOESM1_ESM.docx]

**Additional file**

**Tables**

**Table 1:** Concentrations of minor chemicals present in growth media constructed using oilcane hydrolysate and juice blends prior and post fermentation.

| *S. cerevisiae* strains | Growth media | Acetic acid (g/l) | | Furfurals (g/l) | | HMF (g/l) | | Glycerol  (g/l) | Xylitol  (g/l) |
| --- | --- | --- | --- | --- | --- | --- | --- | --- | --- |
|  |  | Before | After | Before | After | Before | After |  |  |
| CT2-Pro | Undiluted Hydrolysate | 5.61 ± 0.02 | 3.29 ± 0.77 | 0.15 ± 0.01 | 0.02 ± 0.005 | BD^1^ | BD | BD | BD |
|  | 50:50  Hydrolysate: Juice | 3.05 ± 0.03 | 2.92 ± 0.04 | BD | BD | BD | BD | BD | BD |
| NKSW7-1 | Undiluted Hydrolysate | 6.15 ± 0.02 | 6.73 ± 0.01 | 0.01 ± 0.001 | 0.01 ± 0.001 | 0.14 ± 0.01 | 0.13 ± 0.01 | ND^2^ | ND |
|  | 50:50  Hydrolysate: Juice | 4.67 ± 0.01 | 2.39 ± 0.01 | 0.03 ±0.01 | 0.02 ± 0.005 | BD | BD | BD | 0.31 ± 0.01 |
| Commercial strain | Undiluted Hydrolysate | 4.82 ± 0.05 | 4.58 ± 0.11 | 0.15 ± 0.01 | 0.14 ± 0.01 | 0.19 ± 0.01 | 0.02 ± 0.01 | ND | ND |
|  | 50:50 Hydrolysate: Juice | 3.66 ± 0.27 | 1.63 ± 0.25 | 0.04 ± 0.01 | 0.03 ± 0.01 | BD | BD | BD | BD |

^1^Below Detectable limit.

^2^Not Determined for NKSW7-1 and commercial yeast strains growing on undiluted hydrolysate because the cultures did not grow.

**Table 2 a):** Phospholipids in oilcane hydrolysate and juice obtained using untargeted LC-MS metabolite analysis. The results are reported as signal's peak area counts normalized to the internal standard's peak area count and/or sample volume/sample weight. b) LC/MS analysis of lipid classes, lipid ions and ion formulae of ten major lipids in hydrolysates and juice derived from transgenic oilcane.

**a)**

| **b)LipidIon** | **Oilcane-Juice** | **Oilcane-Hydrolysate** |
| --- | --- | --- |
| LBPA(14:0_14:0)+NH4 | 91695928 | 67015660 |
| LPC(24:0)+H | 7051315 | 4306733 |
| PA(14:0_14:0)-H | 919878.4807 | 889357 |
| PA(16:0_18:1)-H | 10204336.97 | 0 |
| PA(16:0_18:2)-H | 15298544.67 | 0 |
| PA(18:2_18:2)-H | 4186252.712 | 0 |
| PC(32:0)+H | 6796222 | 1848653 |
| PC(34:1)+H | 94664670 | 303029 |
| PC(34:2)+H | 132855520 | 179760 |
| PC(34:3)+H | 8221556 | 0 |
| PC(36:3)+H | 33161393 | 59203 |
| PC(36:4)+H | 35022159 | 77554 |
| PC(46:0)+H | 116328966 | 88473500 |
| PC(50:3)+H | 423997959 | 335202580 |
| PE(26:0)+H | 3043625 | 5452482 |
| PE(30:3)+H | 182267771 | 95392861 |
| PE(34:2)+H | 30881889 | 0 |
| PG(12:0_14:0)-H | 2787015.121 | 3022973 |

b)

| **Lipid class** | **Fatty acid** | **Ion formula** |
| --- | --- | --- |
| Triglyceride | (16:0_14:0_14:0) | C47 H94 O6 N1 |
| Triglyceride | (16:0_16:0_17:0) | C52 H104 O6 N1 |
| Triglyceride | (16:0_16:0_24:0) | C59 H118 O6 N1 |
| Triglyceride | (16:0_16:1_24:0) | C59 H116 O6 N1 |
| Triglyceride | (16:0_17:0_18:1) | C54 H106 O6 N1 |
| Triglyceride | (16:0_18:1_24:0) | C61 H120 O6 N1 |
| Triglyceride | (18:0_18:0_18:1) | C57 H112 O6 N1 |
| Triglyceride | (18:0_18:1_18:1) | C57 H110 O6 N1 |
| Diglyceride | (18:0_18:0) | C39 H80 O5 N1 |
| Diglyceride | (36:2e) | C39 H75 O4 |

**Table 3:** Growth kinetics of xylose metabolizing S. cerevisiae strains in aerobic cultures without acclimated strains. The medium was an equal blend of hydrolysate and juice.

| *S. cerevisiae* strains | Total Sugar concentration (g/l) | Ethanol titer  (g/l) | Biomass yield  (g/g) | Ethanol yield  (g/g) | SEP^1^  (g/g^.^h) | VEP^2^  (g/l^.^h) | SSC^3^ rate (g/g^.^h) |
| --- | --- | --- | --- | --- | --- | --- | --- |
| CT2-Pro | 115.88 ± 0.41 | 20.36 ± 0.01.709 | 0.101 ± 0.01 | 0.38 ± 0.002 | 0.052 ± 0.004 | 0.282 ± 0.023 | 0.138 ± 0.012 |
|  | 69.60 ± 0.01 | 16.49 ± 0.01 | 0.111 ± 0.001 | 0.46 ± 0.002 | 0.174 ± 0.004 | 0.687 ± 0.003 | 0.373 ± 0.003 |
| NKSW7-1 | 115.88 ± 0.41 | 0 | ND^1^ | ND | ND | ND | ND |
|  | 69.60 ± 0.01 | 0 | ND | ND | ND | ND | ND |
| Commercial strain | 115.88 ± 0.41 | 0 | ND | ND | ND | ND | ND |
|  | 69.60 ± 0.01 | 19.73 ± 3.08 | 0.084 ± 0.018 | 0.37 ± 0.002 | 0.049 ± 0.015 | 0.205 ± 0.032 | 0.129 ± 0.028 |

^1^Specific ethanol productivity

^2^Volumetric ethanol productivity

^3^Specific Sugar Consumption

^4^Not Determined because the yeast culture did not grow.

**Figures**

**Fig. S 1:** Sugar consumption profile of metabolically engineered S. cerevisiae strains CT2-Pro (a, b), NKSW7-1 (c, d) and commercial yeast (e, f) on 100% hydrolysate (a, c, e) and 50:50 Hydrolysate: Juice (b, d, f) derived from transgenic oilcane,

**Fig. S 2:** Sugar consumption profile of metabolically engineered S. cerevisiae strains CT2-Pro (a, b), NKSW7-1 (c, d) and commercial yeast (e, f). The strains were acclimated on 50:50 Hydrolysate: Juice media prior to fermentation. Hydrolysate: Juice 50:50 media was diluted to get a total sugar concentration of ~110 g/l (a, c and e) and ~75 g/l (b, d and f).

**Fig. S 3:** Growth and fermentation profile of metabolically engineered S. cerevisiae strains CT2-Pro (a, b), NKSW7-1 (c, d) and commercial yeast (e, f). The strains were not acclimated prior to fermentation. Hydrolysate: Juice 50:50 media was diluted to get a total sugar concentration of ~110 g/l (a, c and e) and ~75 g/l (b, d and f).
